# Baicalein improves motor dysfunction and cognitive impairment while promoting remyelination in an animal model of multiple sclerosis through the antioxidant mechanism

**DOI:** 10.3389/fphar.2025.1659631

**Published:** 2025-09-10

**Authors:** Qin Wang, Ziwen Wang, Yutong Li, Qiongzhang Wang, Yanran Chen, Benhao Xu, Haiyun Xu

**Affiliations:** ^1^ School of Mental Health, Wenzhou Medical University; Wenzhou Key Laboratory of Basic and Translational Research for Mental Disorders, Wenzhou, Zhejiang, China; ^2^ The Affiliated Kangning Hospital, Wenzhou Medical University, Wenzhou, Zhejiang, China; ^3^ Department of Neurology, The First Affiliated Hospital, Wenzhou Medical University, Wenzhou, Zhejiang, China

**Keywords:** baicalein, cuprizone, demyelination, mitochondria, oligodendrocytes, remyelination

## Abstract

**Background:**

Multiple sclerosis (MS) is a chronic inflammatory and neurodegenerative disease in which repetitive demyelination defeats remyelination and impairs axonal conduction thus resulting in the characteristic disabilities of MS. In searching for new drugs to treat MS, traditional Chinese herbs are gaining increasing attention.

**Methods:**

In animal experiments, an animal model of MS was established by administering cuprizone (CPZ, a copper chelator toxic to cell mitochondria) to C57BL/6J male mice. The therapeutic effects of baicalein (BA) were comprehensively investigated by examining its effects on the CPZ induced neuropathological changes and behavioral abnormalities. Moreover, the cellular and molecular mechanisms underlying the therapeutic effects of BA were explored. The *in vitro* experiments were done with cultured oligodendrocyte (OL) lineage cells and OLN-93 cell line at various conditions of the absence or presence of CPZ, H_2_O_2,_ and BA. The viability, development, and mitochondrial function of the cultured cells, as well as oxidative stress measures in the cells were analyzed by means of cell biological and biochemical methods.

**Results:**

In the *in vivo* experiments, BA facilitated the recovery of motor and cognitive impairment in CPZ-exposed mice while promoting the remyelination process and inhibiting neuroinflammation in their brains. Underlying these protective effects, BA prevented the nuclear factor erythroid 2-related factor 2 (NRF2) and its downstream antioxidant enzymes (HO-1, NQO1, and SOD2) from over-activation, thereby maintaining the signal pathway in normal levels through its antioxidant actions. The *in vitro* experiments provided evidence that both CPZ and H_2_O_2_ delay the development of oligodendrocyte (OL) lineage cells by damaging mitochondria of the cells and resulting in oxidative stress. BA effectively prevented cultured OLs from development delay by scavenging ROS resulting from damaged mitochondria of the cells.

**Conclusion:**

These data demonstrate that BA facilitates remyelination and development of OL lineage cells in the MS model of CPZ-exposed mouse by its antioxidant actions thereby encouraging future clinical applications of BA in treating patients with MS.

## Graphical Abstract

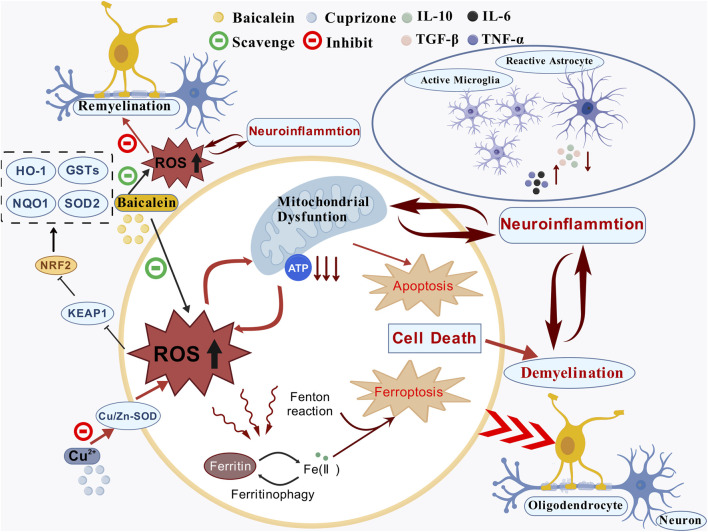

## 1 Background

Multiple sclerosis (MS) is a chronic inflammatory and neurodegenerative disease with primary neuropathological changes of demyelination, neuroinflammation, gliosis, and neurodegeneration in the central nervous system (CNS) (Tobore, 2020). MS patients may have diverse clinical manifestations including motor dysfunction and cognitive impairment such as reduced information processing speed, and impaired attention and long-term episodic memory (Woo et al., 2024). Approximately 2.8 million people worldwide are affected by MS, with young adults aged 20–30 years being the most susceptible population (Walton et al., 2020). It is believed that the pathogenesis of MS involves T-lymphocytes-mediated inflammation leading to the inflammation of gray and white matter, along with myelin sheath destruction and impaired remyelination (Tanaka et al., 2020; Zarghami et al., 2021), in which mitochondrial dysfunction and oxidative stress play important roles (Bargiela and Chinnery, 2019; Corthay, 2006; Gambini and Stromsnes, 2022; McGarry et al., 2018). Related to the T-lymphocytes-mediated inflammation in MS, experimental autoimmune encephalomyelitis (EAE) is the most commonly used experimental model for MS. In line with the mitochondrial dysfunction and oxidative stress (OS) in MS, the dietary consumption of cuprizone (CPZ, a copper chelator toxic to mitochondria of cells) has been known to induce demyelination and subsequent remyelination in the mouse brain, thus being used as another animal model of MS (Abdel-Maged et al., 2020; Zirngibl et al., 2022).

Mitochondria are highly reactive to various cellular signals, including OS, and play a crucial role in regulating apoptosis (Signorile et al., 2020). Mitochondrial dysfunction has been observed in both immune cells and oligodendrocytes (OLs) in MS patients (López-Muguruza and Matute, 2023). In mice exposed to CPZ, mitochondria in OLs were most susceptible to the toxic effects of this chemical, whereas astrocytes and microglia were activated (Luo et al., 2020; Yang et al., 2020b). These previous data indicate that neuroinflammation and OS are inextricably linked in the pathogenesis of MS. Indeed, glial and infiltrated immune cells are considered major producers of reactive oxygen species (ROS) and reactive nitrogen species (RNS) in pathological conditions of the CNS (D'Ambrosi et al., 2018).

Since 1993, when the FDA approved IFN-β1b for the treatment of relapsing forms of MS (Paty and Li, 1993), a growing number of therapies have changed the management of the disease considerably, but patients have to face increasing and sometimes even severe risks from side effects of the active disease-modifying therapies. For example, long-term follow-up of patients in a pivotal trial of intramuscular IFN-β1a revealed a poor prognosis for patients who exhibited active radiographic disease during the 2 years on the drug (Bermel and Inglese, 2012). MS patients treated with natalizumab (Tysabri, a monoclonal antibody targeting the α4 integrin) presented progressive multifocal leukoencephalopathy (PML) being the major side effect while inhibiting the inflammatory aspects of MS (Langer-Gould et al., 2005; Morrow et al., 2022; Polman, 2006; Rudick et al., 2006). The other second-era drug, fingolimod, was reported to suppress MS disease activity, with 55%–60% lower relapse rates and an impressive reduction of MRI-visible activity (Cohen et al., 2010; He et al., 2015; Kappos et al., 2010; Pelletier and Hafler, 2012), but it may leads to a side effect of lymphopenia associated with reactivation of varicella zoster virus (VCV) (Tanaka, 2016).

In the search for new drugs to treat MS, antioxidant compounds are gaining interest. A recent literature review summarized roles of naturally derived or combined derivatives of natural compounds, with well-known antioxidant potential, in MS progression modulation (Theodosis-Nobelos and Rekka, 2022). Evidence suggests that these natural compounds exert considerable role in MS pathogenesis by slowing down its progression or improving some of its clinical manifestations, such as fatigue, cognitive dysfunction and paralysis, along with substantial decreases in many oxidative and inflammatory markers. These previous studies encouraged further research on antioxidant compounds or naturally derived compounds, with multi-targeting and disease modulating potential, for the establishment of new treatment or co-treatment approaches in MS.

Baicalein is a flavonoid extract derived from the roots of *Scutellaria baicalensis* Georgi (SBG). It exhibits strong neuroprotective effects but has no toxic effect in the broad range of tested doses. The root of SBG is a classic compatible component in the decoction of herbal medicine used for treating CNS diseases. Previous studies reported the association of SBG consumption with a lower incidence of dementia and other age-related neurological disorders (Gasiorowski et al., 2011). Moreover, BA has been shown to scavenge superoxide and hydroxyl radicals and alleviate mitochondrial dysfunction by activating the transcription factor nuclear factor erythroid 2-related factor 2 (NRF2) and mediating the production of the antioxidant enzyme Mn-SOD in previous studies (Licht-Mayer et al., 2015). In mice with EAE, BA significantly attenuated clinical severity of EAE and inhibited migration of autoimmune T cells into the CNS (Xu J. et al., 2013). In CPZ-exposed mice, BA alleviated CPZ-induced demyelination, glial activation, pro-inflammatory cytokine expression, and motor dysfunction, suggesting that BA may be used as a useful therapeutic agent in demyelinating diseases such as MS (Hashimoto et al., 2017).

Based on the previous studies, we hypothesized that BA might protect OLs against the toxic effects of CPZ on the development of the OL lineage cells by its antioxidant action thereby promoting the remyelination process in CPZ-exposed mice. To test this hypothesis, experiments with C57BL/6J mice and cultured OLs were done. In the animal experiments, we examined the effects of BA on remyelination and OL development in CPZ-exposed mice, evaluated its potential in promoting the recovery process of the mice from behavioral abnormalities induced by CPZ exposure, and explored the neurobiological mechanisms underlying its protective effects. In cultured OLs and OLN-93 cells, we demonstrated that BA blocks the inhibitory effects of CPZ and H_2_O_2_ on the development of OL lineage cells by protecting mitochondrial function of the cells.

## 2 Materials and methods

### 2.1 Reagents and chemicals

Cuprizone (purity >99%) and D-biotin were purchased from Sigma (St. Louis, Missouri, United States). B27 Plus was from Thermo Fisher (Waltham, Massachusetts, United States). ELISA kits for the assessment of IL-6, TNF-α, IL-10, TGF-β, NRF2, HO-1, NQO1, and SOD2 were from Keduo Di Biotechnology (Fujian, China). Fetal bovine serum (FBS), Dulbecco’s Modified Eagle Medium (DMEM), DMEM/F-12, penicillin/streptomycin (P/S) solution and Accutase were purchased from Gibco (Carlsbad, California, United States). b-FGF and PDGF-AA were purchased from Peprotech (New Jersey, United States). Bovine serum albumin (BSA), Triton X-100, JC-1, CCK8, and ROS assay kits were from Beyotime Biotechnology (Shanghai, China). Sterile PBS buffer, sodium citrate repair solution, RIPA lysis buffer, corticosterone and triiodothyronine were purchased from Solarbio (Beijing, China). PVDF membrane was purchased from Millipore (Billerica, Massachusetts, United States). BCA protein assay kit and ECL exposure solution were purchased from NCM Biotech (Suzhou, China). The suppliers and catalog numbers of all primary and second antibodies used in this study are presented in Table 1.

**TABLE 1:** The suppliers and catalog numbers of antibodies used in the study.

Antibodies	Suppliers	Catalog number
Mouse anti-IBA1	Abcam	ab178846
Mouse anti-Olig2	Abcam	ab109186
Mouse anti-OXPHOS	Abcam	ab110413
Rabbit anti-APC	Abcam	ab40778
Rabbit anti-MBP	Abcam	ab40390
Rabbit anti-MOG	Abcam	ab32760
Goat anti-rabbit antibody Alexa Fluor 488	Abcam	ab150077
Mouse anti-GAPDH	Proteintech	60004-1
Rabbit anti-NAT8L-	Proteintech	23841-1-AP
Mouse anti-GFAP-	Proteintech	60190-1
HRP-conjugated goat anti-mouse	Proteintech	SA00001-1
HRP-conjugated goat anti-rabbit	Proteintech	SA00001-2
Mouse anti-Olig4	Sigma	O7139
Goat anti-mouse antibody Alexa Fluor 488	Thermo Fisher	A-11001
Goat anti-rabbit antibody Alexa Fluor 594	Thermo Fisher	A-11012

### 2.2 Experimental design and animal groups

This study consists of experiments with C57BL/6J male mice and cultured OL lineage cells. The C57BL/6J male mice in 7 weeks old were provided by Zhejiang Viton Lihua laboratory Animal Technology Co., Ltd. After an adaptation period of 7 days, all mice were randomly assigned into each of the following five groups: Control group (CNT), in which mice were fed standard mouse chow for 6 weeks without any additional intervention; CPZ+S group, in which mice were fed standard mouse chow containing 0.2% (w/w) cuprizone for 4 weeks, followed by administration of sterile saline (0.9%, gavage feeding at 1.0 mL/100 g body weight) for 2 weeks; and the other three groups in which mice were given BA at 10, 20, or 40 mg/kg in the same volume (1.0 mL/100 g body weight) of sterile saline as that in CPZ+S group. These three groups were referred to as CPZ+BA10, CPZ+BA20, or CPZ+BA40, respectively. By referring to a previous animal study (Li Y. et al., 2021), we chose the above doses of BA in this study. The CPZ+BA10 group consisted of 12 mice in two batches, each of the other four groups consisted of 17 mice in three batches.

After behavioral tests, mice of each group were euthanized and their brains were subjected to the further analyses of immunofluorescence staining, Western blotting, and ELISA, in three subgroups, each of 5 or 6. In the data analyses, outliers that have a value exceeding 1.5 SD of the group average were prevented from further statistical analysis. This manipulation led to the uneven numbers in different analyses for the same animal groups.

The *in vitro* experiments were done with primary OL cultures or OLN-93 cell line, a rat OPC cell line obtained from BeNa Culture Collection (Hebei, China). For primary OL cultures, pregnant SD rats (Zhejiang Viton Lihua laboratory Animal Technology Co., Ltd.) were used.

The mice and pregnant SD rats were maintained in the SPF-grade animal facility of the School of Mental Health, Wenzhou Medical University. They were group-housed (six mice per cage) with free accesses to water and food. The animal facility was well-ventilated, with a constant temperature (22 °C ± 1 °C), in a humidity range of 50%–60%, and a 12 h/12 h light-dark cycle. All animal experimental procedures described in this study were following the guidelines on animal care and use of Wenzhou Medical University and approved by the Ethics Committee of Wenzhou Medical University (2023Y07M07D). All animal experiments followed the 3Rs principle, and animals suffering was minimized as much as possible throughout the experimental procedures.

### 2.3 Behavioral tests

#### 2.3.1 Rotarod test

This is a classic test for evaluating motor coordination in rodents. Before the test, the starting speed of the rotarod device (YLS-4D, Nuolei Xinda Technology Co., Ltd., Tianjin, China) was set to 5 r/min, with a uniform accelerating speed from 5 r/min to 50 r/min within 6 min. When tested, a mouse was placed on the rotarod with his forepaws attached to the horizontal bar. The time from the start of the test until the first time the mouse fell off the rod or turned around on the rod was recorded. If a mouse did not slip or flip over for more than 5 min, the first flip latency was recorded as 5 min. The experiment was repeated two times for each mouse at an interval of 15 min, and the average value of the two latencies was finally used to evaluate the motor coordination ability of the mouse. After each experiment, the rotarod was wiped clean with 75% alcohol to eliminate the odor from a previous mouse.

#### 2.3.2 Y-maze test

The Y-maze consisted of three identical arms radiating outward from the joint area at an identical angle of 120 ° between two arms. All arms had the same dimensions of 30 cm (length), 8 cm (width), and 15 cm (height). Mice were acclimated to the behavioral laboratory environment for 1 day before the test. During the test, a mouse was placed at the end of an arm and allowed to freely explore the Y-maze for 8 min, during which period the mouse’s movement trajectory on the maze was recorded by a camera above the maze. After a test, the interior walls of the maze were cleaned with 75% alcohol to eliminate the odor from the tested mouse. Mice with normal spatial memory are expected to visit the three arms in a non-repetitive order, such as ABC, BCA, CAB, and so on. The total number of arm entries was recorded, and the percentage of the actual number of correct arm entries to the theoretical number of correct arm entries (total number of arm entries minus 2) is referred to as the spontaneous alternation rate (expressed as percentage).

#### 2.3.3 Novel object recognition (NOR) test

The protocol described here was modified from that by Ennaceur and Delacour (Ennaceur and Delacour, 1988). The test was performed in a gray open-top cube empty box (with dimensions of 45 cm in length, width, and height) in 3 days. The apparatus was placed in a sound-isolated room.

The NOR procedure consisted of the habituation, training, and retention trials in 3 days. On the first day, mouse was habituated to the apparatus by exploring the test area in the absence of objects for 5 min. To avoid the presence of olfactory trails, the apparatus after each trial was thoroughly cleaned. On the second day, mouse was individually put back into the box and allowed to explore the same test area in the presence of two identical objects for 10 min (training trial). Exploration of an object was defined as directing the nose to the object at a distance of maximum 1 cm and/or touching it with the nose. The objects could not be displaced by a mouse as they were stuck to the wall of the open field. After a 2 h rest period, the same mouse was subjected to another test (first retention trial) in which he explored two objects, a familiar one used in the training trial and a novel one on the same area for 5 min. On the third day, each mouse was subjected to the second retention trial as the first retention trial (exploring a familiar object and a novel one for 5 min). The order of objects used per subject per trial was determined randomly. All combinations and locations of objects were used in a balanced manner to reduce potential biases because of preferences for particular locations or objects.

The exploration time for the novel and familiar objects in the retention trials was recorded, and the recognition index (RI) was quantified as the preference for the novel object, with the formula: RI = (time exploring the novel object)/(time exploring the novel object + time exploring the familiar object).

### 2.4 Immunofluorescence staining

For brain tissue immunofluorescence staining (IFS), the mouse was anesthetized with 1.25% tribromoethanol at a dose of 2.0 mL/100 g body weight. Under the deep anesthesia, the mouse was transcardially perfused with the phosphate buffered saline (PBS, pH = 7.4) until the liquid flowing out of the right atrial appendage was clear and the liver turned white. Then the whole brain of mouse was removed out of the skull and post-fixed in 4% paraformaldehyde (PFA) at 4 °C for 3 days and subsequently transferred into 30% sucrose solution at 4 °C for 48–72 h. The brain samples were then cut into 30-µm-thickness cross-sections using a Leica cryostat (CM30505, Leica Microsystems, Wetzlar, Germany) for IFS. The procedures for IFS of the brain sections include: 1) brain sections were fixed in 4% PFA for 30 min, then placed in a 1 × sodium citrate solution at 95 °C for 30 min; 2) after cooling to room temperature, the sections were incubated with a mixture of 5% BSA and 0.3% Triton X-100 for 60 min at room temperature; 3) one of primary antibodies (Rabbit anti-MBP, 1:1,000; Mouse anti-Olig2, 1:500; Mouse anti-GFAP, 1:500; Rabbit anti-IBA-1, 1:1,000) was applied and incubated overnight at 4 °C in a humidified chamber; 4) After rinsing with PBS 3 × 5 min, a second antibody (Goat anti-mouse secondary antibody Alexa Fluor 488, 1:1,000; Goat anti-rabbit secondary antibody Alexa Fluor 488, 1:1,000; or Goat anti-rabbit secondary antibody Alexa Fluor 594, 1:1,000) was applied and incubated for 60 min at room temperature; 5) the sections were mounted with a DAPI-containing mounting medium, dried, and observed under an inverted fluorescence microscope for imaging (LEICA DMI8, Wetzlar, Germany). The IFS images were analyzed using the ImageJ software (NIH, Bethesda, MD, United States).

The procedures of IFS for cultured oligodendrocyte linage cells include: 1) the slides with cultured cells (OLs) were rinsed with PBS 3 × 5 min; 2) 4% PFA was added onto the slides to fix the cells for 15 min, followed by PBS rinsing three times; 3) the slides with OLs were immersed in 0.3% Triton X-100 for 10 min × 3; 4) the slides were immersed in 5% BSA for 60 min; 5) the slides were incubated with a primary antibody (Mouse anti-Olig2, 1:200; Mouse anti-Olig4, 1:200; Rabbit anti-APC, 1:500) at 4 °C overnight; 6) after rinsing with PBS 3 times, the slides were incubated with a secondary antibody specific to a primary antibody for 60 min at room temperature; 7) after rinsing with PBS 3 times, the slides were sealed with a sealing agent containing DAPI; 8) observed under an inverted fluorescence microscope for imaging (LEICA DMI8, Wetzlar, Germany). The IFS images were analyzed using the ImageJ software.

### 2.5 Western blotting analysis

Tissue samples from the prefrontal cortex (PFC) and hippocampus (HIP) of mice were weighed and lysed with RIPA lysis buffer (1.0 mg in 1.0 mL), homogenized at 4 °C, and centrifuged at 12,000 rpm for 15 min. The supernatant was collected, and protein concentration was measured. Equal amounts of protein (20 μg) were loaded onto each well of the 4% stacking gel and run onto the 10% SDS-polyacrylamide gel. Then the proteins were transferred from the separating gel to a PVDF membrane. The membrane was blocked with 5% skim milk for 1.5 h and incubated with a primary antibody overnight at 4 °C. The primary antibodies used for the Western blotting experiments include rabbit anti-MBP (1:1,000), rabbit anti-MOG (1:1,000), mouse anti-total OXPHOS cocktail (1:250), mouse anti-GFAP (1:500), and rabbit anti-NAT8L (1:2,000), with the mouse anti-GAPDH (1:10,000) as the internal control. After rinsing the PVDF membrane with 1 × TBST (3 × 10 min), the membrane was incubated with a secondary antibody of horseradish peroxidase-conjugated goat anti-rabbit (1:10,000) or horseradish peroxidase-conjugated goat anti-mouse (1:10,000) for 1 h at room temperature. Finally, the membrane was developed using ECL exposure solution in a gel imaging system (VILBER, Paris, France), and the bands were quantified using ImageJ software.

### 2.6 Enzyme-linked immunosorbent assay (ELISA) of cytokines

Tissue samples from the PFC and HIP of mice were weighed and homogenized in PBS (100 mg tissue added into 900 µL PBS). The homogenate was centrifuged at 3,000 rpm, 4 °C, for 20 min and the supernatant was collected. 50 μL of the prepared samples and standards were added to each well of an ELISA plate, followed by the addition of 50 µL biotinylated antigen working solution and incubated at 37 °C for 30 min. After spilling out the solutions, all wells were rinsed 5 times with PBS, then 50 µL of streptavidin-HRP working solution was added into each well and incubated at 37 °C for 30 min. After rinsing wells with PBS again, color development solutions A and B (50 µL each) were added into each well and incubated at 37 °C for 10 min. The reaction was stopped by adding 50 µL of stop solution, and the OD value was read out within 10 min at 450 nm using an ELISA microplate reader (TECAN Infinite M200 Pro, Switzerland). Protein levels of the inflammatory cytokines including IL-6, TNF-α, IL-10, and TGF-β were calculated using ELISA calc with a logistic curve (four-parameter) model.

### 2.7 Cell culture of primary oligodendrocytes and drug treatments

By referring to a previous study (Xu H. et al., 2013), brain cells in cerebral cortex of newborn SD rats (0–2 days old) were extracted and seeded at a density of 1 × 10^6^/cm^2^ onto poly-D-lysine-coated T75 culture flasks and incubated at 37 °C in a CO_2_ incubator. The medium was changed every 3 days until day 9 *in vitro* (DIV9). The T75 flasks were placed on a shaker at 150 rpm for 60 min to separate microglial cells. Subsequently, the flasks were shaken continuously at 250 rpm for 15–20 h to separate oligodendrocyte precursor cells (OPCs) from astrocytes. The medium was then transferred to culture dishes and allowed to settle for 60 min to remove adherent astrocytes and microglia. Then the medium was centrifuged at 1,800 rpm for 6 min, and the pellets were resuspended and seeded onto cell culture inserts or slides at an appropriate density. After culturing in proliferation medium (DMEM supplemented with 2% B27 Plus, 0.5% FBS, 1% P/S, 0.1% PDGF-AA, and 0.1% b-FGF) for 2 days, the cells were switched to differentiation medium (DMEM/F12 was supplemented with 0.5% FBS, 1% P/S, 1% nitrogen, 10 nM Corticosterone, 10 nM D-biotin, and 30 nM triiodothyronine) for further culturing.

In this experiment, OPCs were exposed to 0.5 mM CPZ for 24 h starting from DIV11. Differentiated OLs were exposed to CPZ for 24 h starting from DIV13. Immature OLs were exposed to CPZ for 24 h starting from DIV15.

### 2.8 Cell culture of OLN-93 cell line

OLN-93, a rat OPC cell line, was obtained from BeNa Culture Collection (Hebei, China). The cells were cultured in DMEM (supplemented with 10% FBS, 1% P/S) at 37 °C in a humidified atmosphere containing 95% air and 5% CO_2_. Cells at 80%–90% confluence were selected for subculture and subsequent experimentation.

### 2.9 CCK-8 assay

WST-8 (Cell Counting Kit 8; CCK-8) has been accepted as a validated method for measuring the viability of cultured cells. It can be reduced by dehydrogenases in the mitochondria to produce an orange-yellow formazan in the presence of an electron coupling reagent. The color intensity is proportional to cell viability. In our experiment, extracted OL linage cells were cultured in 96-well plates until approximately 80% of the well area is covered by the cells. Then various concentrations of CPZ (0, 0.025, 0.05, 0.1, 0.5, 1.0, 2.0 mM) were added into the culture medium of primary OLs on DIV11-12, or various concentrations of H_2_O_2_ (0.2, 0.4, 0.8, or 1.6 mM) were added into the culture medium of OLN-93 cells when the cell density reaches 80%–90%. Both the culture conditions maintained for 24 h. Then the culture medium was removed, and the cells were washed once with PBS, followed by the addition of 100 µL fresh medium and 10 µL CCK-8 solution into each well. Furthermore, the plate was incubated in a CO_2_ incubator for additional 2 h before absorbance measurement at 450 nm on the same microplate reader as mentioned above. Each group was prepared in 6 wells at least 3 independent experiments. The relative cell viability was expressed as a percentage of the control cells that were treated neither with CPZ nor with H_2_O_2_.

### 2.10 Assessment of mitochondrial membrane potential

During the early stage of differentiation (DIV13-DIV14), primary OPCs were treated with CPZ (0, 0.5 mM) and BA (0, 20 μM (Kim et al., 2023)) for 24 h, then the culture medium was removed from the confocal culture dishes, and the cells were washed once with PBS and then stained by a mixture solution consisting of equal volume (each of 1.0 mL) of culture medium and JC-1 staining working solution at 37 °C for 20 min. After incubation, the supernatant was removed, and the cells were washed twice with JC-1 staining buffer. Then 2.0 mL of culture medium was added, and the cells were observed under a laser confocal microscope (LEICA DMI8, Wetzlar, Germany). The relative degrees of mitochondrial polarization were quantified by measuring the red-shifted JC-1 at 535 nm (Ex) and 590 (Em) and green shifted JC-1 at 515 nm (Ex) and 529 nm (Em).

### 2.11 Detection of ROS

Intracellular ROS can oxidize non-fluorescent DCFH to fluorescent DCF, allowing the detection of ROS levels based on fluorescence intensity. In brief, when the cell density reaches 80%–90%, the OLN-93 cells were treated with H_2_O_2_ (0, 400 μM) and BA (0, 40 μM (Kim et al., 2023)) for 24 h. Then the original culture medium was removed, followed by the addition of diluted DCFH-DA (10 µM/L) into the cell-containing wells. After incubation at 37 °C for 20 min, the cultures were rinsed three times with serum-free culture medium. Finally, the ROS levels were detected using a fluorescence microplate reader (LEICA DMI8, Wetzlar, Germany).

### 2.12 Statistical analysis

SPSS 25 was used to analyze the data. Data conforming to a normal distribution were expressed as mean ± standard error of the mean (Mean ± SEM). Levene’s test was used to assess homogeneity of variance. For data in multiple groups, one-way ANOVA or repeated-measures ANOVA was used to evaluate the effects of experimental treatments, followed by LSD multiple comparisons for group-wise comparisons. For comparisons between two groups, t-tests were used, with *p* < 0.05 indicating statistical significance. GraphPad Prism 9 was used for graphing. Each experiment was repeated at least three times.

## 3 Results

### 3.1 Baicalein improved motor dysfunction and cognitive impairment in cuprizone-exposed mice

**FIGURE 1:**
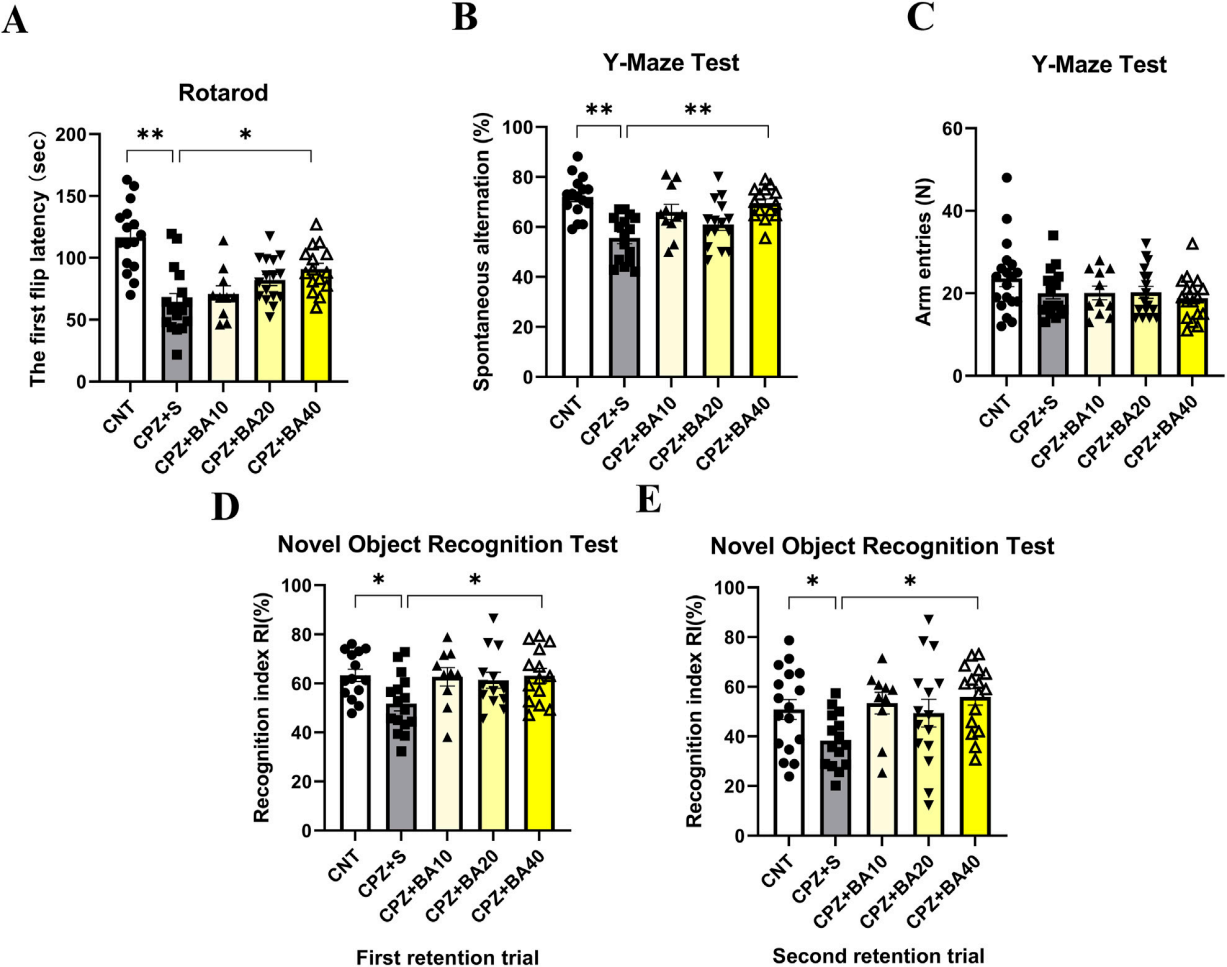
Baicalein alleviates CPZ-induced behavioral changes in mice. **(A)** Results of the rotarod test (n = 10–16/group). **(B,C)** Results of the Y-maze test (n = 11–19/group). **(D,E)** Results of the novel object recognition test (n = 10–17/group); Data are presented as mean ± SEM. *p < 0.05, **p < 0.01.

In the rotarod test, the mice in the five groups performed differently in terms of the first flip latency (Figure 1A). One-way ANOVA of the results indicated a significant main effect of grouping (F = 12.27, p < 0.0001). Post-hoc tests reported a significant difference between CNT and CPZ+S groups in the first flip latency (p < 0.0001). The differences also existed between CNT and CPZ+BA10 (p < 0.0001)/BA20 (p = 0.0005)/BA40 (p = 0.0191) groups. Moreover, the CPZ+BA40 group showed a significantly longer first flip latency relative to the CPZ+S group (p = 0.0159).

In the Y-maze test, the mice in the five groups also performed differently (Figures 1B,C). One-way ANOVA of the spontaneous alternation revealed a significant main effect of grouping (F = 9.295, p < 0.0001). Post-hoc tests reported that the spontaneous alternation in the CPZ + S group was significantly lower than that in the CNT group (p < 0.0001), but no significant difference existed between CNT and CPZ+BA40 groups. Moreover, CPZ+BA40 group exhibited a significant higher spontaneous alternation compared to the CPZ+S group (p = 0.0003). Interestingly, one-way ANOVA of the arm entries data showed no significant effect of grouping.

In the novel object recognition test, mice in all five groups performed differently in terms of RI in the two retention trials. For the first retention trial (done 2 h after the training trial), one-way ANOVA revealed a significant effect of grouping on RI (F = 3.244, p = 0.0298). Post-hoc tests reported a significant lower RI in the CPZ+S group compared to the CNT group (p = 0.0464). It was also significantly lower than that in the CPZ+BA40 group (p = 0.0442). But there was no difference between any other two groups (Figure 1D). Same results were also found in the second retention trial, i.e., the main effect of grouping is significant on RI (F = 3.754, p = 0.0162), significant differences existed between CNT and CPZ+S groups (p = 0.0177), between CPZ+S and CPZ+BA40 groups (p = 0.0129) (Figure 1E).

### 3.2 Baicalein promoted remyelination following CPZ-induced demyelination in mice

**FIGURE 2:**
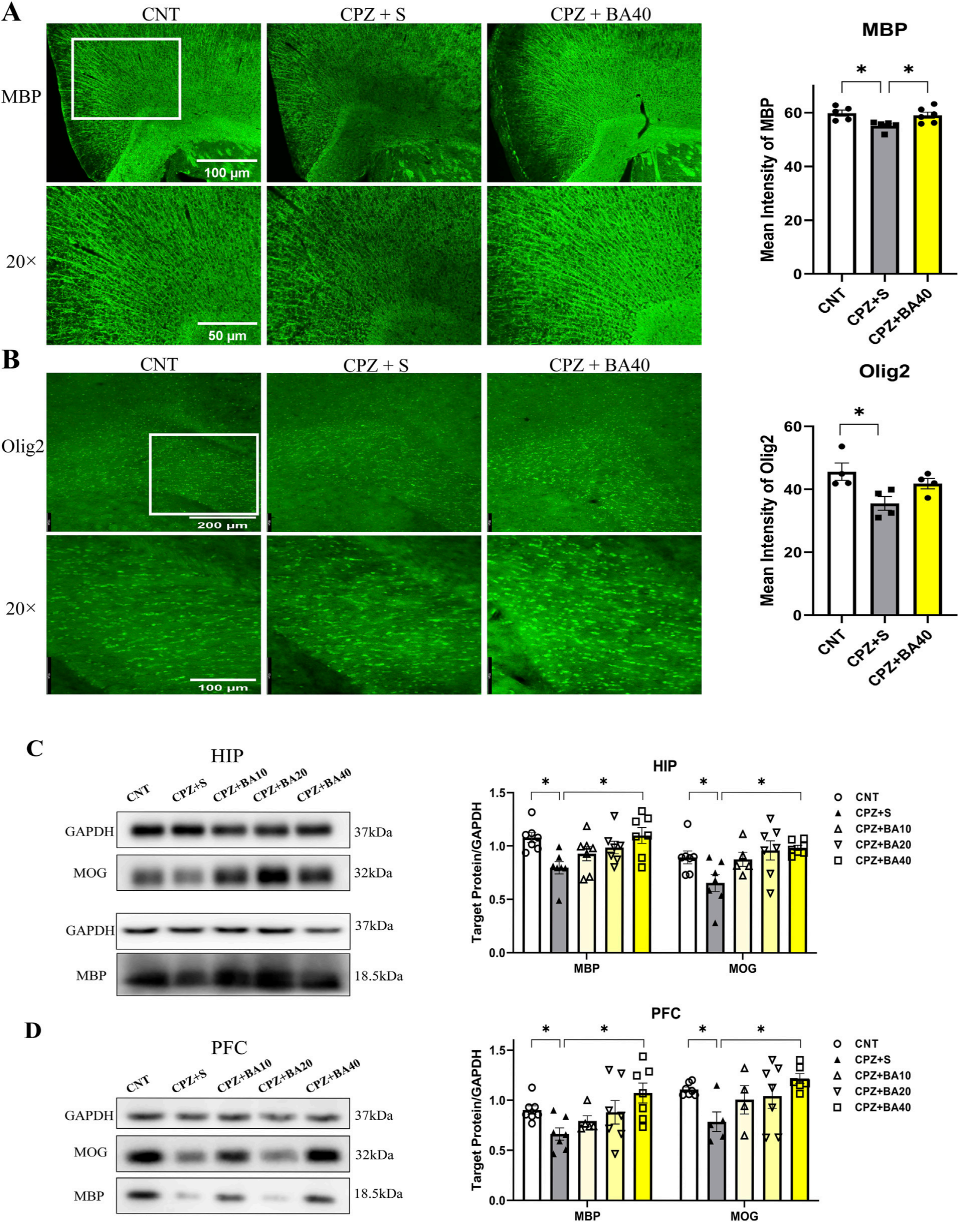
Baicalein promotes remyelination following CPZ-induced demyelination in the mouse brain. **(A)** Representative immunofluorescence staining images of MBP in the cerebral cortex and the statistical analysis of MBP MFI there (n = 5/group). **(B)** Representative immunofluorescence staining images of Olig2 in the corpus callosum and the statistical analysis of Olig2 MFI there (n = 4/group). **(C)** The representative Western blotting images of MBP and MOG proteins in HIP and the quantitative analysis of the proteins there (n = 5–7/group). **(D)** The representative Western blotting images of MBP and MOG proteins in the cerebral cortex and the quantitative analysis of the proteins there (n = 4–7/group). *p < 0.05, data are presented as mean ± SEM.

Figure 2A shows representative MBP IFS images taken at cerebral cortex of the mice from the three groups of CNT, CPZ+S, and CPZ+BA40. Close observation of these images at a higher magnification tells obvious differences in terms of MBP immunostaining in the mice. Consistent with this visual observation, grouping showed a significant effect on the MFI of MBP (F = 5.440, p = 0.0192). Post-hoc comparisons reported that CPZ+S had a significant lower MFI of MBP relative to CNT group (p = 0.0235) or CPZ+BA40 group (p = 0.0482), there was no difference between CNT and CPZ+BA40 groups (p = ns). These results suggest that administration of BA at 40 mg/kg promoted the remyelination process in brains of mice following the CPZ-induced demyelination.

To provide further evidence for the above inference, we measured the MFI of oligodendrocyte transcription factor 2 (Olig2) IFS in the brains of mice from the same three groups. Figure 2B shows representative Olig2 IFS images taken at the corpus callosum (CC). Close observation of these images at a higher magnification tells obvious difference in Olig2 immunostaining in CC. Consistent with this visual observation, quantitative data of Olig2 MFI reported a significant effect of grouping (F = 5.075, p = 0.0334). Post-hoc comparisons showed a significant difference between CPZ+S and CNT groups (p = 0.0285), indicating an inhibiting effect of CPZ exposure on the development of OL lineage cells in this brain region. However, no difference was found between CNT and CPZ+BA40 groups (p = ns), suggesting a protective effect of BA (at the dose of 40 mg/kg) against the toxic effect of CPZ on OL lineage cells.

Further evidence came from the Western blotting analysis of MBP and myelin oligodendrocyte glycoprotein (MOG) in HIP and PFC of mice from all five animal groups. Figure 2C presents the representative Western blotting images of MBP and MOG (GADPH as the internal control), plus the quantitative data of the two proteins in HIP. One-way ANOVA revealed a significant effect of grouping on MBP (F = 4.006, p = 0.0101) and MOG (F = 3.830, p = 0.0132) expression levels. Post-hoc comparisons reported significant lower levels of MBP and MOG in the CPZ+S group relative to the CNT group (p = 0.0223, p = 0.0334, respectively) and to the CPZ+BA40 group (p = 0.0121, p = 0.0128, respectively). There was no significant difference between CNT group and any one of BA-treated groups. Similar changes were also seen in the expression levels of MBP and MOG in PFC of the mice, i.e., there was a significant effect of grouping on MBP (F = 3.428, p = 0.0211) and MOG (F = 2.863, p = 0.0452) expression levels. MBP and MOG levels in the CPZ+S group were significantly lower compared to the CNT group (p = 0.0101, p = 0.0252, respectively) and CPZ+BA40 group (p = 0.0075, p = 0.0037, respectively). There was no significant difference between CNT group and any one of BA-treated groups (Figure 2D).

### 3.3 Baicalein suppressed neuroinflammation in cuprizone-exposed mice

**FIGURE 3:**
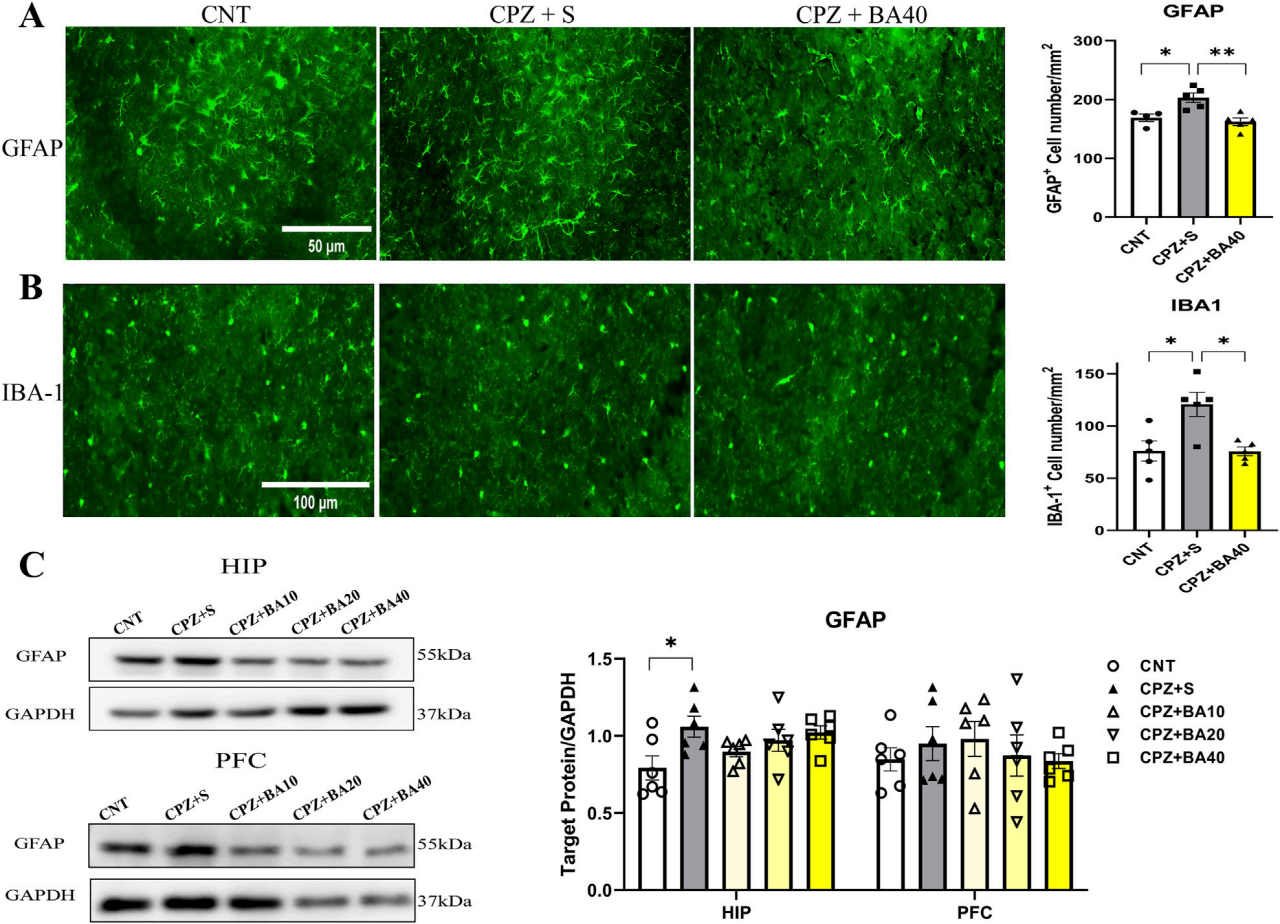
Baicalein alleviates the CPZ-induced proliferation of astrocytes and microglia in the mouse brain. **(A)** Representative immunofluorescence staining images of GFAP in the CA3 region of HIP and statistical analysis of GFAP^+^ cell density there (n = 5/group). **(B)** Representative immunofluorescence staining images of IBA-1^+^ cells in the CA3 region and statistical analysis of IBA-1^+^ cell density there (n = 5/group). **(C)** Representative Western blotting images of GFAP in the HIP and PFC and statistical analysis of GFAP expression in the brain regions (n = 6/group). *p < 0.05, data are presented as mean ± SEM.

We performed IFS on brain sections of mice in the three groups of CNT, CPZ+S, and CPZ+BA40 with the antibodies specific to GFAP and IBA1. Figure 3A shows the representative images of the GFAP^+^ cells in HIP of the mice in these three groups. The GFAP^+^ cells in the CPZ+S image seem to be more densely packed than those in the other two images from CNT and CPZ+BA40 groups, respectively. One-way ANOVA revealed a significant effect of grouping on the density of GFAP^+^ cells in the CA3 region of HIP. Post-hoc multiple comparisons reported that the number of GFAP^+^ cells in the CA3 region of the CPZ+S group was significantly higher compared to those in the CNT group (p = 0.0175) and those in the CPZ+BA40 group (p = 0.0038). Similar changes were also found in IBA1^+^ cells. The IBA1^+^ cells in the CPZ+S image seem to be more densely packed than those in the other two images from CNT and CPZ+BA40 groups, respectively (Figure 3B). One-way ANOVA revealed a significant effect of grouping on the density of IBA1^+^ cells in the CA3 region of HIP. Post-hoc comparisons reported that the number of IBA1^+^ cells in the CA3 region of the CPZ+S group was significantly higher compared to those in the CNT group (p = 0.0112) and those in the CPZ+BA40 group (p = 0.0104).

To provide further evidence for the aforementioned IFS findings, we assessed the expression levels of GFAP protein in the HIP and PFC of mice from all five groups (n = 6/group) by means of Western blotting. Figure 3C shows representative Western blotting images of GFAP protein expressed in HIP and PFC, plus the statistical analysis results of the Western blotting data. One-way ANOVA revealed a significant effect of grouping on GFAP expression levels in HIP (F = 2.972, P < 0.05). Post-hoc tests reported that the GFAP expression level in the CPZ+S group was significantly higher than that in the CNT group. There was no significant difference between any other two groups. For GFAP expression level in PFC, one-way ANOVA showed no significant effect of grouping on this measurement (F = 1.540, p = 0.235).

**FIGURE 4:**
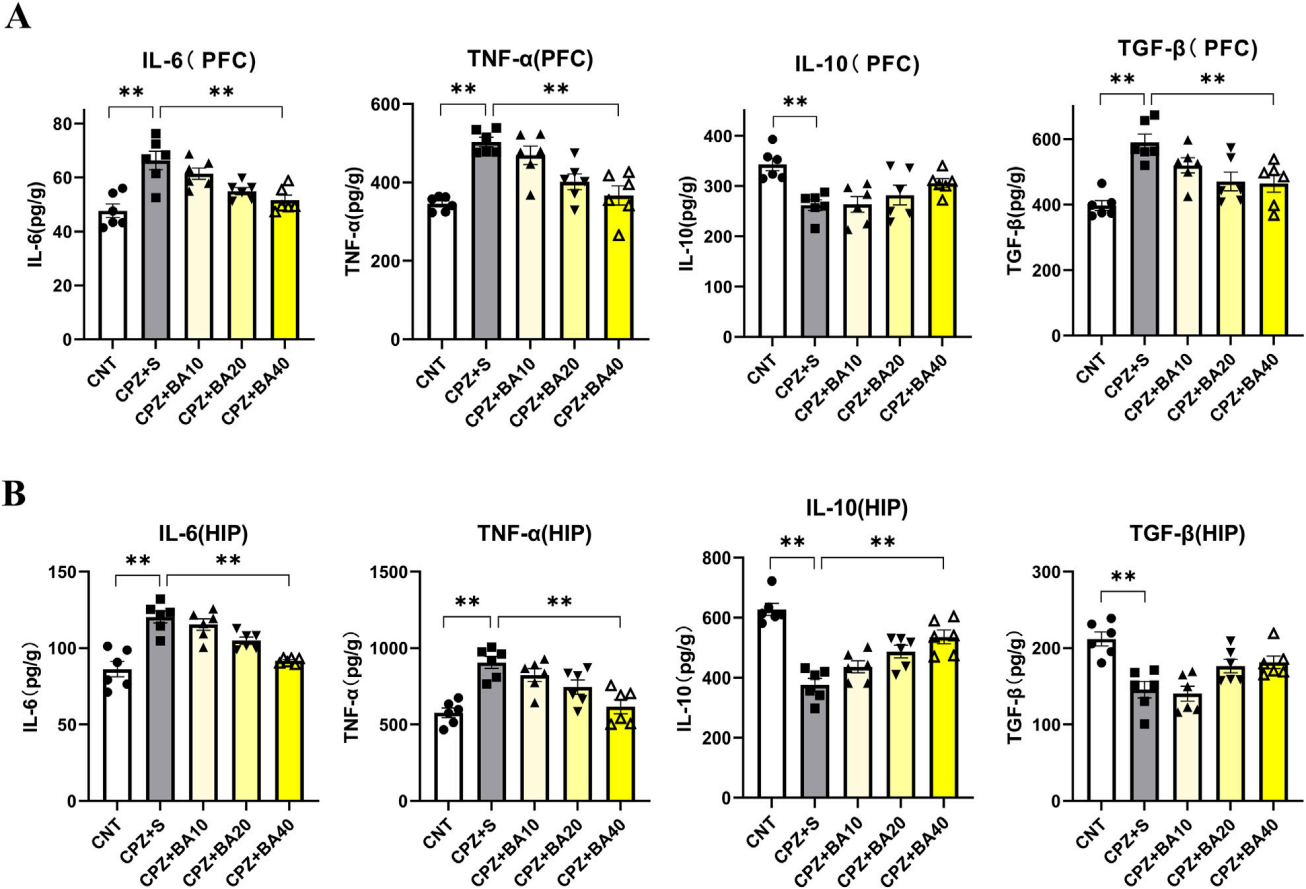
Baicalein reverses the CPZ-induced changes in levels of inflammatory cytokines in the mouse brain. **(A)** Bar charts showing the levels of IL-6, TNF-α, IL-10 and TGF-β in the PFC (n = 6/group). **(B)** Bar charts showing the levels of IL-6, TNF-α, IL-10 and TGF-β in the HIP (n = 6/group). *p < 0.05, **p < 0.01. Data are presented as mean ± SEM.

The proliferation and activation of microglia and/or astrocytes are always accompanied by changes in levels of inflammatory cytokines in the brain. Therefore, we quantitatively assessed the protein levels of pro-inflammatory cytokines (IL-6, TNF-α) and anti-inflammatory cytokines (IL-10, TGF-β) in the PFC and HIP regions of mice from each of the five animal groups. The results are presented in Figure 4. In PFC, one-way ANOVA showed significant effects of grouping on IL-6 (F = 10.170, p < 0.0001) and TNF-α (F = 12.840, p < 0.0001) levels. Post-hoc tests reported that IL-6 and TNF-α levels in the CPZ+S group were significantly higher than those in the CNT group (p < 0.0001 in both comparisons). The levels of these two cytokines were also significantly higher relative to those in CPZ+BA40 group (p = 0.0014, p = 0.0002, respectively). There was no difference between any other two groups in levels of these two inflammatory cytokines. As for IL-10 in PFC, one-way ANOVA showed a significant effect of grouping on this measure (F = 5.917, p = 0.0017). Post-hoc tests reported that IL-10 level in the CPZ+S group was significantly lower than that in the CNT group (p = 0.0032). There was no difference between the other any two groups in this index. Also, one-way ANOVA showed a significant effect of grouping on TGF-β level (F = 8.764, p = 0.0001) in PFC. Post-hoc tests reported that TGF-β level in the CPZ+S group was significantly higher than those in the CNT and CPZ+BA40 groups (p < 0.0001, p = 0.0095, respectively). There was no difference between the other any two groups in this measure (Figure 4A).

Similar changes in these inflammatory cytokines happened in the HIP region. One-way ANOVA showed significant effects of grouping on IL-6 (F = 17.370, p < 0.0001) and TNF-α (F = 11.070, p < 0.0001) levels. Post-hoc tests reported that IL-6 and TNF-α levels in the CPZ+S group were significantly higher than those in the CNT group (p < 0.0001 in both comparisons). The levels of these two cytokines were also significantly higher relative to those in CPZ+BA40 group (p < 0.001 in both comparisons). There was no difference between the other any two groups in levels of these two inflammatory cytokines. As for IL-10 in HIP, one-way ANOVA showed a significant effect of grouping on this measure (F = 20.750, p < 0.0001). Post-hoc tests reported that IL-10 level in the CPZ+S group was significantly lower than those in the CNT and CPZ+BA40 groups (p < 0.001 in both comparisons). There was no difference between the other any two groups in this index. Also, one-way ANOVA showed a significant effect of grouping on TGF-β level (F = 9.655, p < 0.0001) in HIP. Post-hoc tests reported that TGF-β level in CNT group was the highest among the five groups. There was no difference among the other four groups in this measure (Figure 4B).

### 3.4 Baicalein improved mitochondrial dysfunction in CPZ-exposed mice

**FIGURE 5:**
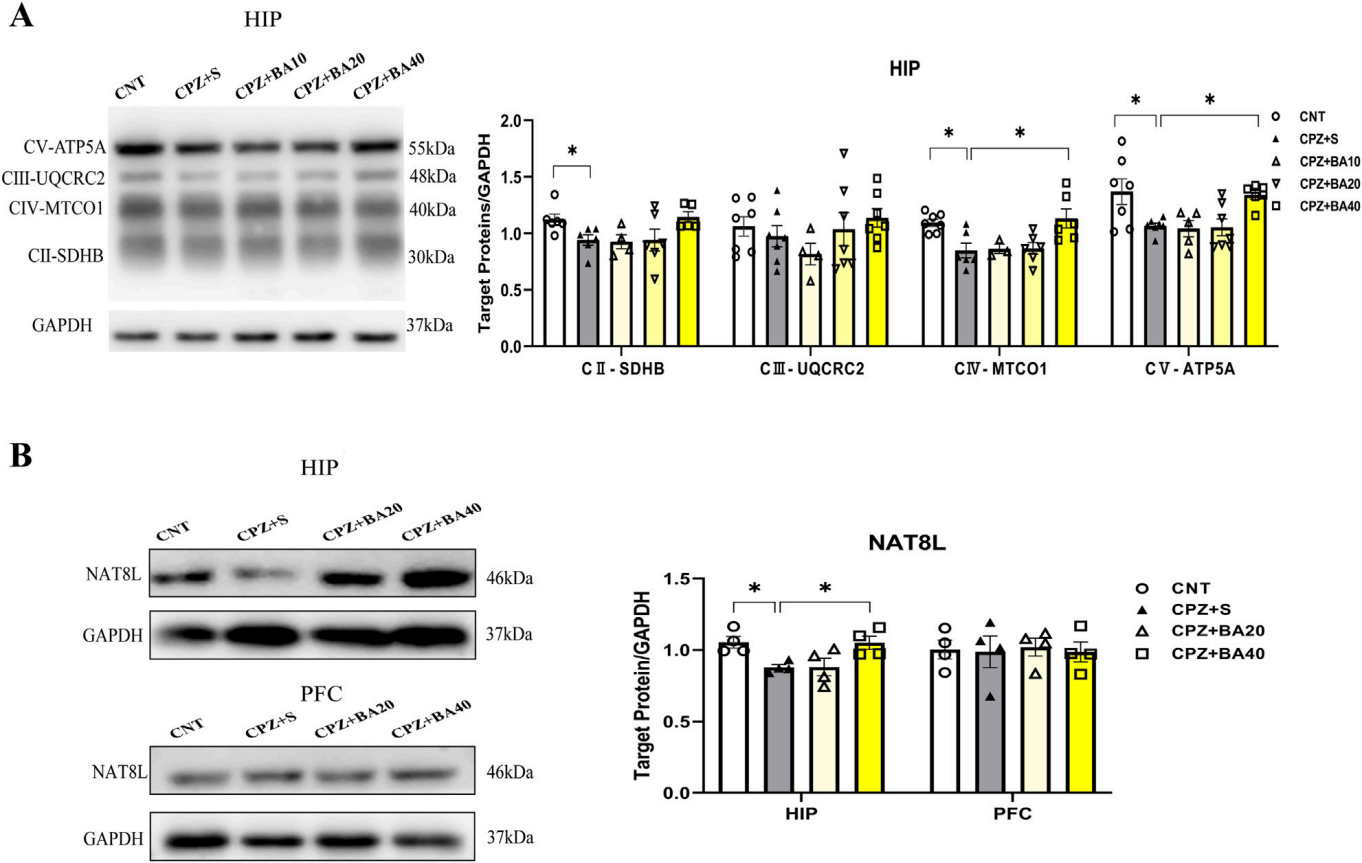
Baicalein facilitates the recovery of decreased mitochondrial respiratory chain complexes in HIP tissue of the CPZ-exposed mice. **(A)** Representative Western blotting images of mitochondrial respiratory chain complexes CII-CV and quantitative analysis of them in the HIP (n = 3–7). **(B)** Representative Western blotting images of the NAT8L protein in the HIP and PFC regions and the quantitative analysis of NAT8L in the two brain regions (n = 4). *p < 0.05, data are presented as mean ± SEM.

Previous studies have shown that CPZ impairs mitochondrial function of neural cells thereby causing demyelination in CNS of mice (Faizi et al., 2016; Luo et al., 2020). We expected that baicalein might protect brain cells against the toxic effects of CPZ on mitochondrial function in mice. To test this hypothesis, we assessed mitochondrial function of neural cells in HIP of all mice in this study by means of Western blotting. Figure 5A shows representative Western blotting images of mitochondrial respiratory chain complexes CII-CV of neural cells of mice from each group (CI was not shown). The quantitative data of all images were presented in Figure 5A (right). One-way ANOVA revealed significant effects of grouping on protein levels of CIV (F = 5.461, p = 0.0031) and CV (F = 4.190, p = 0.004). The grouping effect was at a marginal level on CII (p = 0.058). Post-hoc comparisons reported that CII level in CPZ S group was significantly lower compared to the CNT group (p = 0.0234), there was no difference between CNT and CPZ+BA40 groups (p = ns). The CIV and CV levels in CPZ+S group were significantly lower than those in CNT (p = 0.0394, p = 0.041, respectively) and CPZ+BA40 groups (p = 0.0181, p = 0.0458, respectively).

Furthermore, we assessed the protein levels of NAT8L (aspartate N-acetyltrasferase) in HIP and PFC of mice in the four groups (CPZ+BA10 was not included). NAT8L is a mitochondrial protein that catalyzes the synthesis of N-acetylaspartate (NAA) from aspartate and acetyl-CoA in mitochondria of neuron. The representative Western blotting images of NAT8L in HIP and PFC of mice were presented on the upper and lower rows of the left panel of Figure 5B, respectively, while the statistical analysis of the protein levels in the two brain regions of mice is shown in the right panel of this figure. One-way ANOVA revealed a significant effect of grouping on NAT8L levels in HIP (F = 4.956, p = 0.0183), but not on that in PFC (F = 0.38, p = ns). Post-hoc comparisons reported that NAT8L level in HIP of mice from the CPZ+S group was significantly lower than those in the CNT and CPZ+BA40 groups (p = 0.0433, p = 0.0468, respectively).

### 3.5 Baicalein regulated the NRF2 redox pathway in CPZ-exposed mice

**FIGURE 6:**
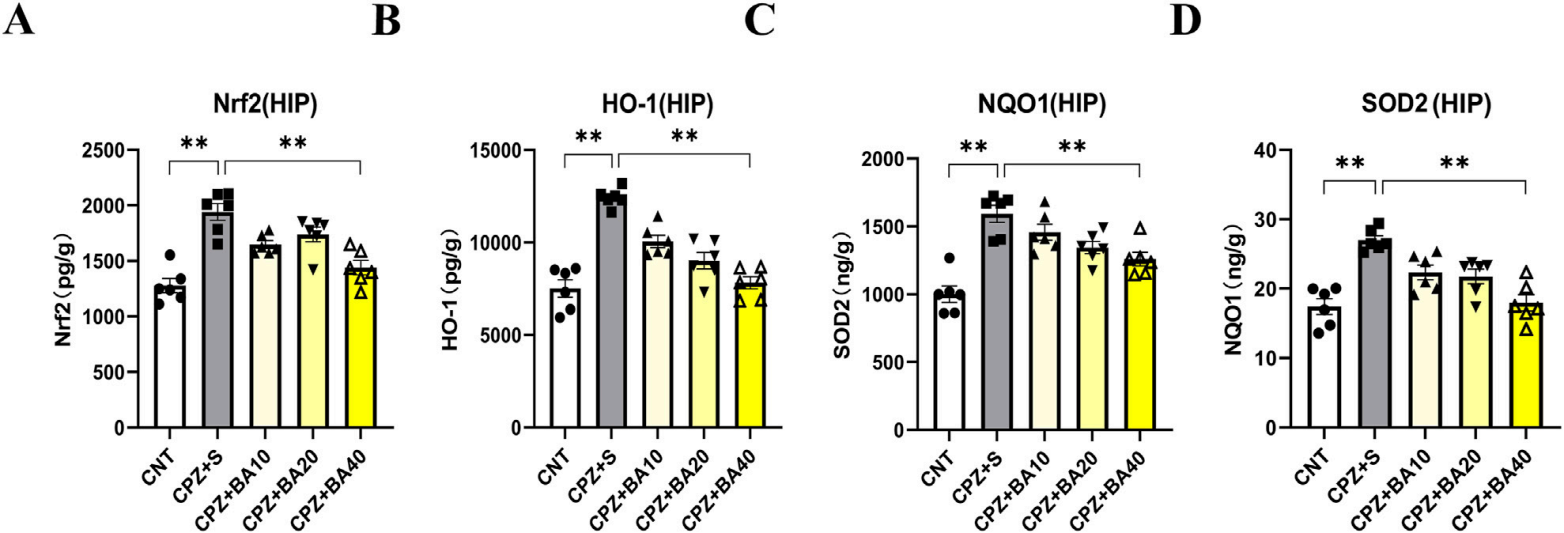
Baicalein facilitates the recovery of the CPZ-induced changes in the NRF2 pathway proteins in HIP of Mice. **(A)** The bar chart showing the quantitative analysis of the NRF2 levels in all mice of the five groups (n = 6/group). **(B)** The bar chart showing the quantitative analysis of the HO-1 levels (n = 6/group). **(C)** The bar chart showing the quantitative analysis of the NQO1 levels (n = 6/group). **(D)** The bar chart showing the quantitative analysis of the SOD2 levels (n = 6/group). *p < 0.05, **p < 0.01. Data are presented as mean ± SEM.

Oxidative stress and mitochondrial dysfunction in MS (Kim et al., 2015) are partly attributed to the imbalance of the NRF2 signaling pathway, which regulates redox homeostasis and mitochondrial function and plays a crucial role in modulating neuroinflammation (Branca et al., 2017; Brandes and Gray, 2020; Chen et al., 2009; MichaliČKovÁ et al., 2020). To explore the mechanism underlying the beneficial effects of BA on remyelination following CPZ-induced demyelination in mice, we measured the expression levels of NRF2 and its downstream targets (HO-1, NQO1, and SOD2) in the HIP of mice by means of ELISA. The results are presented in Figure 6. One-way ANOVA revealed a significant effect of grouping on NRF2 levels (F = 17.080, p < 0.0001). Post-hoc comparisons reported that NRF2 level in CPZ+S group was significantly higher than those in CNT and CPZ+BA40 groups (p < 0.0001 in both comparisons) (Figure 6A). The same changes are also seen in levels of HO-1, SOD2, and NQO1. That is, levels of these downstream molecules of the NRF2 signaling pathway in CPZ+S group are significantly higher than those in CNT and CPZ+BA40 groups (Figures 6B–D, respectively).

### 3.6 Baicalein protected against the inhibitory effects of CPZ on the development of OL lineage cells

**FIGURE 7:**
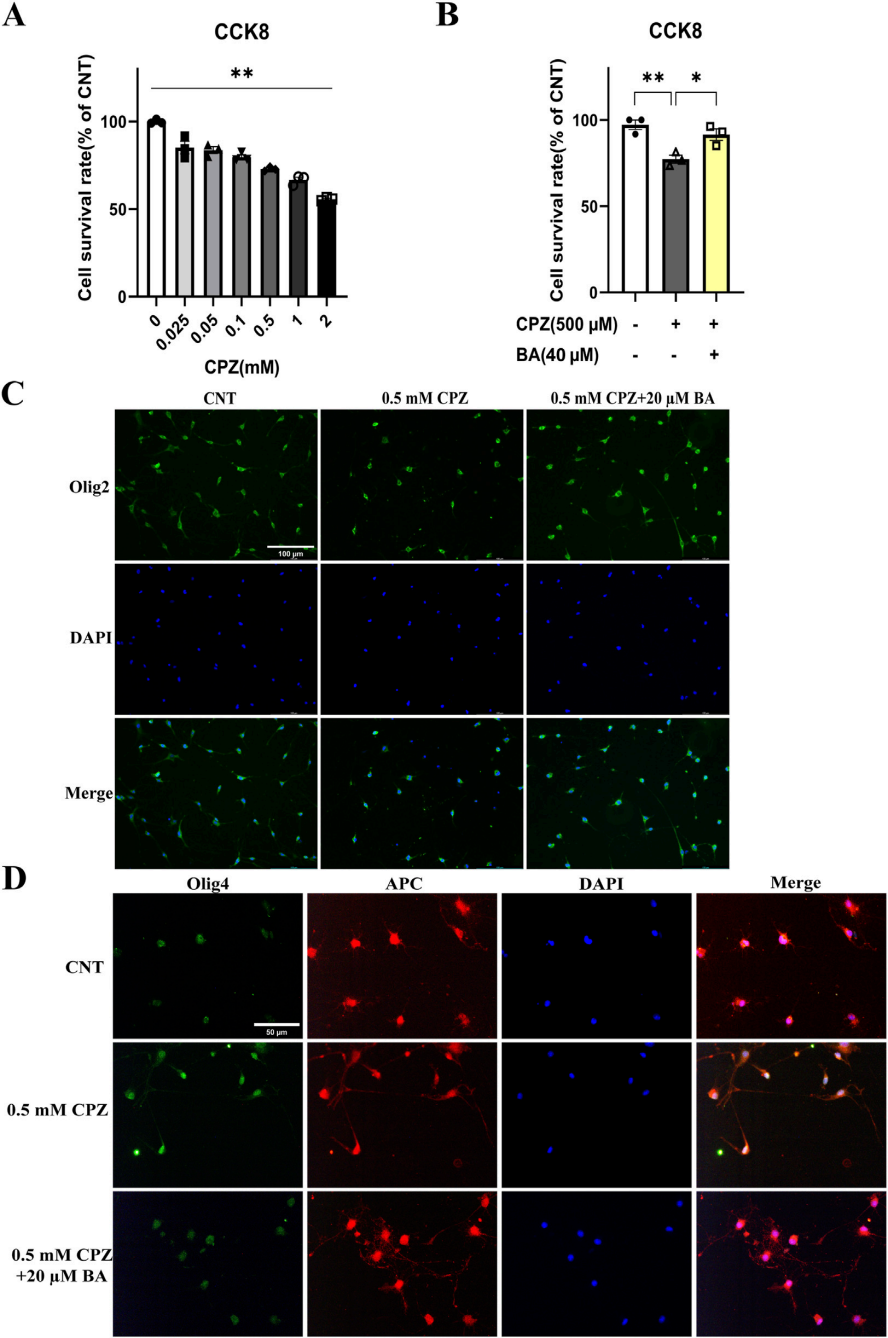
Baicalein blocks the inhibitory effects of CPZ on the viability and development of cultured OL lineage cells. **(A)** CPZ decreases the viability of cultured OPCs in a dose-dependent manner, n = 3/group. **(B)** BA (40 µM) blocks the inhibitory effect of CPZ on the viability of cultured OPCs, n = 3/group. **(C)** Representative immunofluorescence staining images of Olig2^+^ cells cultured under the indicated conditions. **(D)** Representative immunofluorescence staining images of the cells expressing both APC (red) and Olig4 under the indicated conditions. The cell nuclei were labeled by DAPI (blue). *p < 0.05, **p < 0.01. Data are presented as mean ± SEM.

To provide further evidence that BA protects against inhibitory effects of CPZ on the development of OL lineage cells, we did *in vitro* experiments with cultured OLs. During the OPC proliferation phase (DIV11-12), we treated the cells with various concentrations of CPZ (0, 0.025, 0.05, 0.1, 0.5, 1.0, 2.0 mM) for 24 h and measured OPCs viability using the CCK8 assay kit. The quantitative results are shown in Figure 7A. One-way ANOVA revealed a significant main effect of CPZ on OPCs viability (F = 62.920, p < 0.0001). Post-hoc comparisons reported that CPZ dose-dependently reduced OPCs viability as compared to the CNT group. Based on these results, 0.5 mM CPZ and 20 µM BA were chosen to examine possible protective effect of BA on the inhibitory effect of CPZ on OPC proliferation and cell viability. The cells (on DIV11-12) were cultured in three different conditions, i.e., in the absence of CPZ and BA (CNT), in the presence of CPZ (0.5 mM), and in the presence of BA (20 µM) and CPZ (0.5 mM). The results showed a significant main effect of treatment on cell viability (F = 13.300, p = 0.0062). Post-hoc comparisons reported that CPZ-treated cells had significantly lower viability than the CNT (p = 0.0058) and CPZ+BA (p = 0.0276) groups (Figure 7B). In line with the CCK8 assay results, IFS of the cultured cells showed that CPZ group have fewer Olig2^+^ cells relative to the other two groups (see the images on the first row of Figure 7C). And there were many more DAPI-stained nuclei that were smaller (on the second row of Figure 7C) and not overlapped with green-stained cell body and processes (third row of Figure 7C), indicative of condensed nuclei of the dying cells. These results suggest that BA attenuates the inhibitory effects of CPZ on OPCs proliferation and activity.

With the same treatments, another batch of cell cultures started on DIV13 and lasted for 24 h. Then the cultured cells were subjected to IFS with the antibody against Olig4^+^ (a biomarker of immature OLs) or APC^+^ (a biomarker of mature OLs). As shown in Figure 7D, some of the DAPI-stained nuclei on the second row look smaller relative to the others on the first and third rows. In the fourth column with cell images from the superposition of Olig4^+^ and APC^+^, some cell profiles appear pure green in the CPZ (0.5 mM) group, indicating that these immature OLs did not develop to mature ones in the presence of CPZ. This phenomenon was not seen in CNT and CPZ+BA20 groups, indicating that BA attenuated the inhibitory effects of CPZ on the maturation of immature OLs.

### 3.7 Baicalein protected cultured OLs and OLN-93 cells against the toxic effects of CPZ on mitochondria of the cells

**FIGURE 8:**
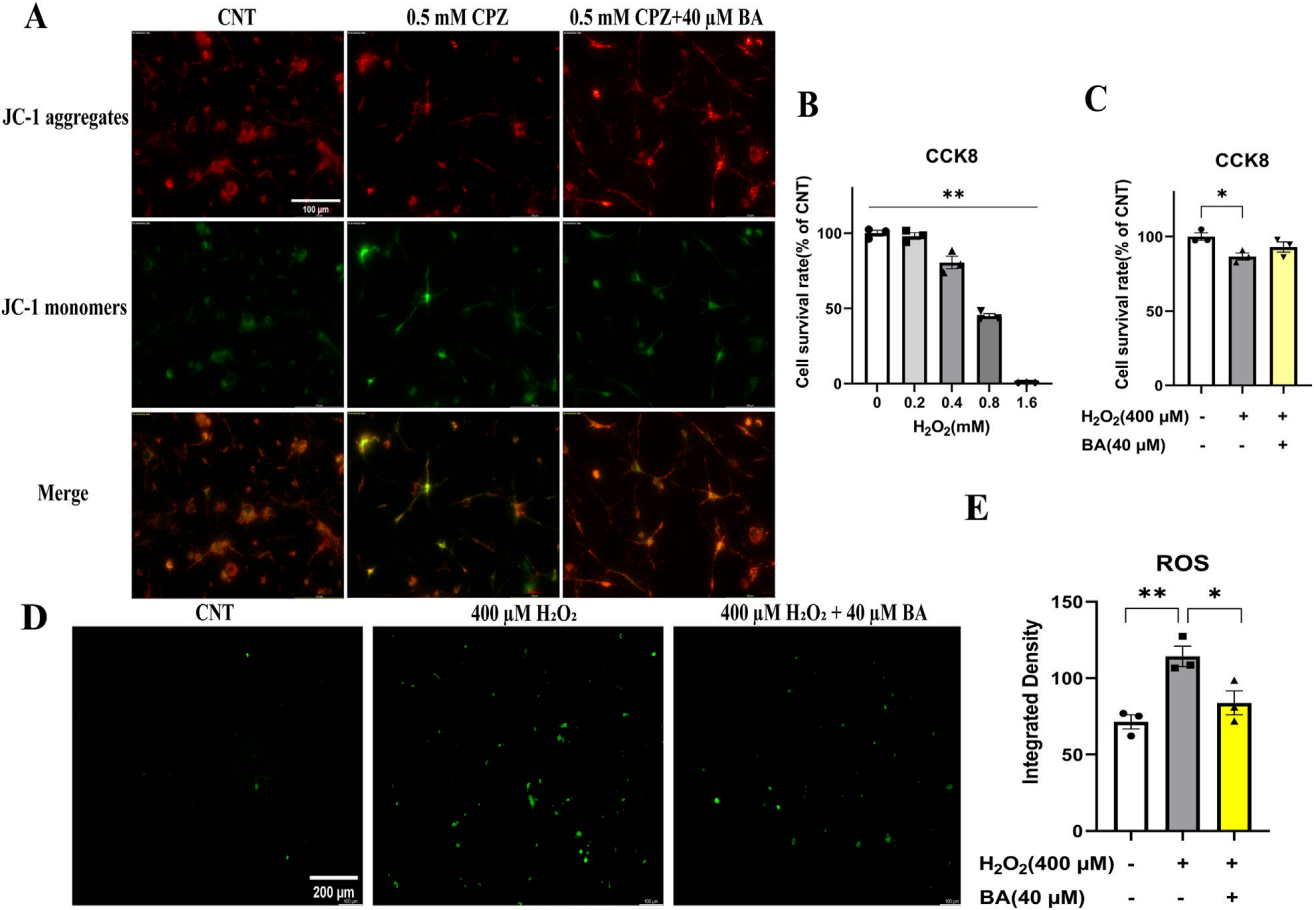
Baicalein prevents cultured OLs from the CPZ-induced mitochondrial membrane potential decrease and blocks the inhibitory effect of H_2_O_2_ on viability of OLN-93 cells by decreasing the production of ROS in the H_2_O_2_-treated cells. **(A)** BA prevents cultured OLs from the CPZ-induced mitochondrial membrane potential decrease. **(B)** H_2_O_2_ decreases the viability of OLN-93 cells in a dose-dependent manner. **(C)** BA prevents OLN-93 cells from H_2_O_2_-induced viability decrease. **(D)** Representative ROS fluorescence images of OLN-93 cells cultured under the indicated conditions, n = 3/group. **(E)** Quantitative analysis of ROS fluorescence intensity in OLN-93 cells cultured under the indicated conditions. *p < 0.05, **p < 0.01. Data are presented as mean ± SEM.

To provide further evidence for the protective effects of baicalein on mitochondrial function of the cultured OLs, we evaluated the mitochondrial membrane potential of OLs cultured under various conditions [in the absence of CPZ and BA, in the presence of CPZ (0.5 mM), or in the presence of CPZ (0.5 mM) + BA (40 µM)]. The culturing lasted for 24 h followed by the assessment of mitochondrial membrane potential of the cultured OLs on DIV13 using the JC-1 kit. As shown in Figure 8A, the CPZ-treated cells appear lighter red fluorescence, but darker green fluorescence, relative to those in the CNT and CPZ+BA groups, indicating the presence of mitochondrial membrane damage in the CPZ-treated cells, which was blocked by BA at the concentration of 40 µM.

Moreover, we did another experiment with the OLN-93 cell line (an immortalized OL cell line) treated with various concentrations of H_2_O_2_ (0, 0.2, 0.4, 0.8, 1.6 mM) for 24 h, followed by cell viability assessment by means of the CCK8 assay. The quantitative results are presented in Figure 8B. One-way ANOVA revealed a significant main effect of H_2_O_2_ concentration on OLN-93 viability (% of CNT). Compared to the CNT group, H_2_O_2_ dose-dependently reduced cell viability of OLN-93. Based on these results, the OLN-93 cell line were cultured in another experiment in the presence of 400 µM H_2_O_2,_ 400 µM H_2_O_2_ plus 40 μM BA, or in the absence of H_2_O_2_ and BA (Control). The culture maintained for 24 h. Then cell viability and ROS levels in the cultures were measured. As shown in Figure 8C, the main effect of treatment on cell viability was significant (F = 5.610, p = 0.0423). Post-hoc comparisons reported a significant difference between CNT and H_2_O_2_ (400 µM) groups. As for ROS levels, OLN-93 cells treated with H_2_O_2_ (400 µM) showed strongest green fluorescence relative to those in the other two groups (Figure 8D). These differences were confirmed by the quantitative analysis of the fluorescence intensity of cultured cells in the three groups (Figure 8E). One-way ANOVA revealed a significant effect of grouping on integrated density of the fluorescence staining of cultured cells (F = 11.510, p = 0.0088). Post-hoc comparisons reported the highest level of fluorescence intensity in the H_2_O_2_ group relative to the CNT (p = 0.0083) and the 400 µM H_2_O_2_+40 µM BA (p = 0.0372) groups.

## 4 Discussion

The pathogenesis of MS is characterized by demyelination, OLs apoptosis, and activation of astrocytes and microglia, ultimately leading to failed remyelination (Podbielska et al., 2013; van der Valk and De Groot, 2000). Demyelination impairs axonal conduction thus resulting in the characteristic disabilities of MS (Sriram, 2011). Therefore, reducing demyelination and promoting remyelination are key strategies for the treatment of MS (Dolati et al., 2017). In searching for new drugs to treat MS, many traditional Chinese herbs are increasingly being studied as complementary and alternative interventions. In the present study, BA was comprehensively investigated regarding its effects on CPZ-induced neuropathological changes and behavioral abnormalities in the CPZ-exposed mice because of its anti-inflammatory, antioxidant, and cognitive-enhancing properties (Brinza et al., 2021; Gregory et al., 2021; Shi et al., 2023). The strong antioxidant properties of BA are partly attributed to the three hydroxyl groups (at positions 5, 6, and 7) of its structure (Supplementary Figure 1), which enable the compound to scavenge ROS through oxidation of these groups (de Oliveira et al., 2015). Additionally, BA can form stable semiquinone radicals, which underpin its potent antioxidant activity (Gao et al., 1999).

Consistent with previous studies from our group and the others (Mihai et al., 2021; Yang et al., 2020a; Zhang et al., 2017), the CPZ-exposed mice in the present study exhibited demyelination in cerebral cortex, HIP, and CC. These mice also showed significantly shorter first flip latency in the rotarod test, lower spontaneous alternation rates in the Y-maze test, and decreased RI in retention trials of the NOR test. These behavioral abnormalities in CPZ-exposed mice suggest the presence of impaired motor coordination and cognitive function resulted from CPZ-exposure, which are reminiscent of the clinical manifestations seen in MS patients as mentioned above. Importantly, BA administration for 2 weeks following 4-weeks CPZ-exposure significantly improved the aforementioned brain function abnormalities while facilitating remyelination process as evidenced by increased MBP and Olig2 IFS along with elevated levels of MBP and MOG in the PFC and HIP of CPZ-exposed mice. Taken together, it is plausible to conclude that BA promoted remyelination in the brain of CPZ-exposed mouse thereby improving motor and cognitive deficits in the animals. This conclusion aligns with the commonly accepted notion that it is demyelination in both white and gray matter in the brain that lead to motor dysfunction and cognitive impairments such as attention deficits and long-term memory loss (Calabrese et al., 2011; Franco-Pons et al., 2007; Hibbits et al., 2009; Kutzelnigg et al., 2005; Woo et al., 2024).

MS patients exhibit pathological features of inflammatory infiltration and glial cell proliferation. Relevantly, the CPZ-induced mice present astrocyte and microglial proliferation and activation, along with increased levels of inflammatory cytokines such as TNF-α and IL-1β. These inflammatory factors act synergistically and impair mitochondrial function of OLs and increase ROS levels as shown in previous studies (Abdel-Maged et al., 2020; Arnett et al., 2001; Pasquini et al., 2006). ROS, in turn, damage mitochondria by blocking complexes in the respiratory chain, leading to OLs apoptosis and ultimately demyelination (Kipp et al., 2009; Mahad et al., 2008; Praet et al., 2014). In consistent with these previous studies, the present study found that CPZ-exposed mice had significantly increased numbers of GFAP^+^ and IBA1^+^ cells in the cerebral cortex and HIP, along with elevated GFAP in HIP, suggesting the presence of neuroinflammation in brain tissue of the CPZ-exposed mice. Further supporting evidence for this inference, the CPZ-exposed mice in this study showed higher levels of pro-inflammatory cytokines TNF-α and IL-6, but significantly lower levels of anti-inflammatory cytokines IL-10 and TGF-β. However, BA given at 40 mg/kg/day for 2 weeks following CPZ-exposure for 4 weeks alleviated the CPZ-induced neuroinflammation, while promoting the remyelination process as discussed above. The concurrent anti-inflammation and promyelinating effects of BA (40 mg/kg/gay for 2 weeks) added further evidence for the application of anti-inflammation drugs in patients with MS. It also highlights the complex interplay between inflammation and demyelination in CNS (Ganz et al., 2023; Zveik et al., 2022; Zveik et al., 2024).

The initial effect of CPZ on neural cells lies in its toxicity to the cells’ mitochondria. Therefore, we examined possible effects of BA on mitochondria of brain cells in CPZ-exposed mice. We found that protein levels of mitochondrial respiratory complexes II, IV, and V (ATP synthase) were significantly lower in the HIP of CPZ-treated mice, along with decreased levels of NAT8L, a marker of neuronal mitochondrial function, indicating the damaging effect of CPZ on mitochondria of both neurons and glia cells. Moreover, BA40 effectively reversed these mitochondria function impairments induced by CPZ-exposure. Further evidence for the damaging effects of CPZ on mitochondrial function of brain cells came from the analysis of the NRF2 pathway, which is a crucial defense mechanism of the cellular response to oxidative stress and mitochondrial dysfunction. The CPZ exposure significantly elevated levels of NRF2 and its down-stream antioxidant enzymes of HO-1, NQO1, and SOD2, confirming the upregulation of this pathway in response to the oxidative stress induced by CPZ exposure. More importantly, BA intervention restored the upregulation of these pathway proteins to normal levels in a dose-dependent manner, suggesting that BA protects the NRF2 pathway from over-activation, maintaining it at normal levels through its antioxidant effects. Certainly, these findings have broad relevance to clinical research advances in MS pathogenesis and treatment. For example, MS patients exhibited mitochondrial dysfunction and impaired mitochondrial respiratory chain complexes, leading to axonal degeneration and demyelination (Campbell and Mahad, 2012; Dutta et al., 2006; Komiyama et al., 2022; Maldonado et al., 2022). Abnormally activated NRF2 pathway was also reported in MS patients (Dinkova-Kostova and Abramov, 2015; Draheim et al., 2016; Licht-Mayer et al., 2015; Rosito et al., 2020). Moreover, the therapeutic effects of the clinical MS drug dimethyl fumarate are believed to be related to this pathway (Uruno and Yamamoto, 2023; Yan et al., 2021).

To provide further evidence for the toxic effects of CPZ on the survival and development of OL lineage cells and the protective effects of BA on these cells, we did *in vitro* experiments with OL lineage cells and OLN-93 cell line. It was found that CPZ dose-dependently decreased the viability of cultured OLs during the OPC proliferation phase (DIV11-12), but this toxic effect was not seen in cultured cells in the presence of BA (20 µM) and CPZ (0.5 mM). This set of data added evidence for the protective effect of BA on OPCs survival and growth in the *in vitro* microenvironment lacking microglia and astrocytes. Going further, we found that CPZ (0.5 mM) suppressed the development of Olig4^+^ cells into more mature APC^+^ cells. And these inhibitory effects were effectively blocked in the CPZ+BA group. In later stages of OL development, CPZ-exposure inhibited the maturation of OLs as some cells stayed at the immature stage (Olig4^+^ cells), but these immature OLs were not seen in the CNT and CPZ+BA groups. Taken together, CPZ suppressed the development of cultured OL lineage cells in the inflammation-free environment, but BA prevented the cultured OL lineage cells from this inhibition of CPZ, thereby allowing the normal growth and development of cultured OL lineage cells.

Last but not least, JC-1 assays revealed that mitochondrial membrane potential of cultured OLs was significantly lower in the CPZ-treated cells compared to the CNT group, but BA (20 µM) blocked this effect. These data added direct evidence that both CPZ and BA impact on mitochondria of cultured OLs in damaging and protecting manners, respectively. Further evidence for the protective effect of BA on mitochondria of cultured OLs came from the experiment with OLN-93 cells treated with various concentrations of H_2_O_2_ in the absence or presence of BA. CCK8 assays reported that H_2_O_2_ dose-dependently reduced the viability of OLN-93 cells, but BA (40 µM) completely blocked this effect. The H_2_O_2_ (400 µM) treatment significantly increased ROS level in the culture medium, but this effect was completely blocked in OLN-93 cells treated H_2_O_2_ and BA (40 µM). Consistent with our results, the H_2_O_2_-induced oxidative stress was reported in a recent study with cultured OLN-93 cells (Li F. et al., 2021). BA has been shown to scavenge free radicals and reduce ROS production in various cell types (Gao et al., 1999; Saija et al., 1995), protect the cells against oxidative damage (Jeong et al., 2019; Ma et al., 2018; Park et al., 2019). All the BA doses used in animal and cell culture experiments in present study are comparable to that used in previous human studies in which 100–2,800 mg/day BA were given to healthy Chinese, showing no significant side effect in the subjects. Therefore, the data from the present study encourage the translational application of BA in clinical practice for MS patients (Dong et al., 2021; Li et al., 2014).

## 5 Conclusion

In conclusion, our *in vivo* experiments demonstrated that BA facilitates the recovery of motor dysfunction and cognitive impairments in CPZ-exposed mice while promoting the remyelination process and inhibiting neuroinflammation in their brains. Underlying these protective effects, BA intervention prevents the NRF2 pathway from over-activation in response to CPZ-exposure, thereby maintaining the signal pathway at relatively normal levels. By damaging mitochondria of the cultured OL lineage cells, both CPZ and H_2_O_2_ delay the development of these cells, whereas BA effectively prevents the cultured OLs from development delay by scavenging ROS resulting from damaged mitochondria of the cells. These sets of *in vivo* and *in vitro* data encourage future clinical application of BA in treating patients with MS.

## Data Availability

All data associated with this study are presented in this manuscript.

## Supplementary material

SUPPLEMENTARY FIGURE 1 The chemical structure of baicalein.
