# Supplementary figures and images for: Baicalein improves motor dysfunction and cognitive impairment while promoting remyelination in an animal model of multiple sclerosis through the antioxidant mechanism
Source: Front Pharmacol. 2025 Sep 10;16:1659631. doi: 10.3389/fphar.2025.1659631 (PMC12457084; doi:10.3389/fphar.2025.1659631)

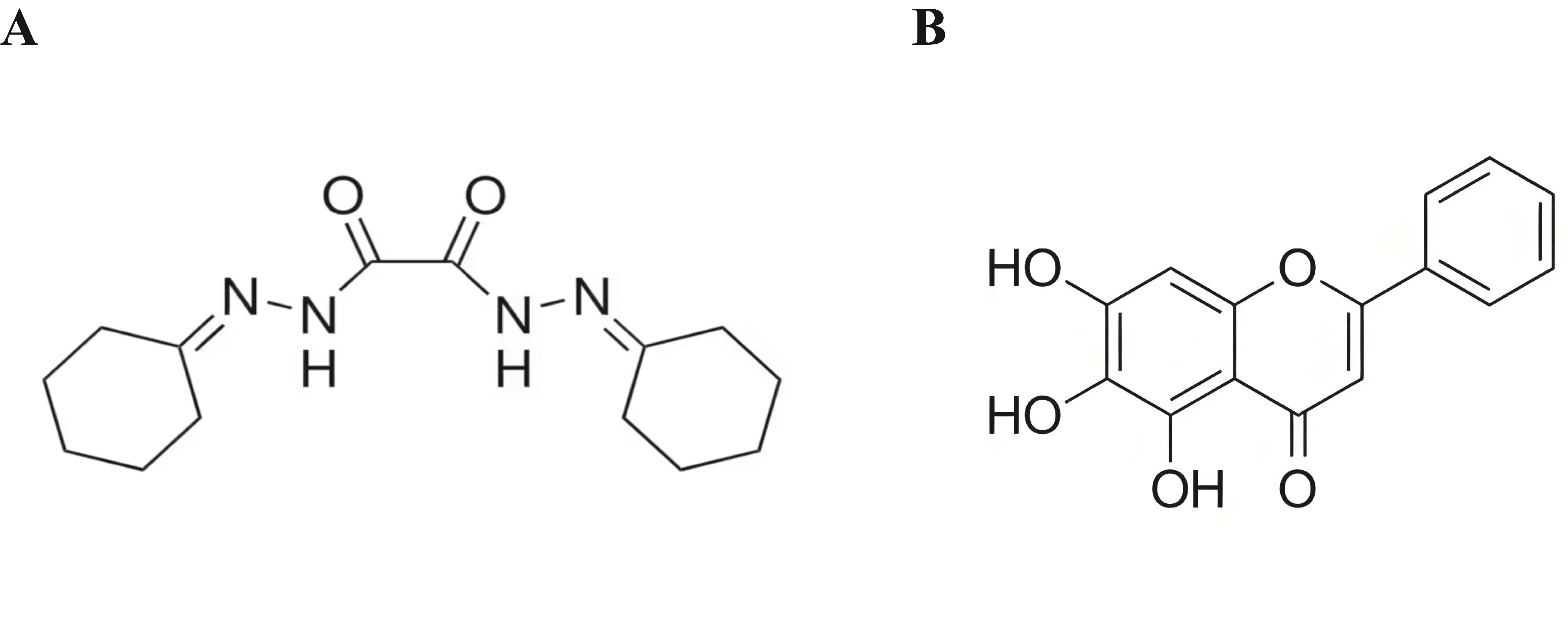

Supplement: Supplementary file 1 [file Image1.jpeg]
